# Hormone replacement therapy and dementia risk among postmenopausal women: Identifying responsive subgroups in the UK Biobank

**DOI:** 10.1002/alz.71679

**Published:** 2026-08-26

**Authors:** Steven Squires, Rasha NM Saleh, Luke C Pilling, Janice L Atkins, Janice M Ranson, Xin You Tai, David Vauzour, David J Llewellyn, Anne‐Marie Minihane

**Affiliations:** ^1^ University of Exeter Medical School Exeter UK; ^2^ Norwich Medical School University of East Anglia Norwich UK; ^3^ Nuffield Department of Clinical Neuroscience University of Oxford Oxford UK; ^4^ Division of Clinical Neurology John Radcliffe Hospital Oxford University Hospitals Trust Oxford UK

**Keywords:** Alzheimer's disease, APOE4, dementia, estrogen, HRT, menopause, precision medicine, surgical menopause

## Abstract

**INTRODUCTION:**

Modifiers of the association between hormone replacement therapy (HRT) use and dementia risk and responsive subgroups, are unknown.

**METHODS:**

In 183,450 postmenopausal women from the UK Biobank (mean 13.3 years follow‐up), multivariable Cox regression models evaluated associations between HRT use (≥1 year) and all‐cause dementia and dementia subtypes. Stratified analyzes assessed modification by menopause type, lifetime estrogen exposure, apolipoprotein E (*APOE*) *ɛ4* genotype, and age at HRT initiation.

**RESULTS:**

Over 2.43 million person‐years, 3,948 dementia cases were identified. HRT use was associated with lower all‐cause dementia (hazard ratio [HR] 0.90; 95% CI, 0.84–0.96), with stronger associations in surgical menopause (0.74, 0.65–0.84), *APOE ɛ4* carriers (0.87, 0.80–0.95) and women with lower endogenous estrogen exposure (0.84, 0.77–0.93). Initiation between ages 46 and 56 conferred the greatest benefit.

**DISCUSSION:**

HRT use was associated with lower dementia risk, and these findings may help inform more tailored approaches to HRT prescribing in relation to women's lifetime dementia risk.

## Highlights

- Hormone replacement therapy (HRT) was associated with lower risk of all‐cause dementia and Alzheimer's disease (AD), but not non‐AD
- Stronger associations were evident for women who had undergone surgical menopause, were apolipoprotein E (*APOE*) *ε4*, and had a shorter time between menarche and menopause
- Initiation of HRT between 46 and 56 years, but not > 56 years, was associated with reduced dementia risk
- While further research is required to establish causality, the findings offer a foundation for future tailored HRT prescribing

## 1 BACKGROUND

Age‐standardized dementia rates are higher in women compared to men, suggesting that female‐specific risk factors may contribute to this disparity.^1^ In premenopausal women, estrogen plays a critical role in supporting brain function through its effects on synaptic integrity, neurotransmitter metabolism, glucose utilization, blood–brain barrier (BBB) maintenance, cerebrovascular function and neuroinflammation.^2^, ^3^ The menopausal transition represents an endocrine inflection point, that often coincides with the onset of the preclinical phase of dementia,^4^ and surgically induced menopause via oophorectomy has been linked with an increased life‐time dementia risk.^5^, ^6^

Despite biological plausibility, evidence regarding the association between hormone replacement therapy (HRT), which is used by approximately 15% of women aged 45–64y in England, and cognitive outcomes or dementia risk is conflicting and inconclusive^7^, ^8^ as highlighted in the most recent meta‐analysis.^9^ The only randomized controlled trial (RCT) to date assessing incident dementia as a primary outcome, the Women's Health Initiative Memory Study (WHIMS, participants ≥ 65 years), reported an increased risk of dementia with combined oral conjugated equine estrogen plus progestin HRT use,^10^ with several other studies concluding similarly^11^, ^12^, ^13^, ^14^ across a diversity of, HRT compositions and routes of delivery, and age of HRT exposure. Conversely, HRT has also been found to be both protective^15^, ^16^, ^17^ (with the Baltimore Longitudinal Study of Ageing looking at estrogen only formulations^15^) and to have no effect in a previous UK Biobank based analyze with shorter follow‐up^18^.

These discrepancies may reflect the influence of multiple modifying factors, many of which remain poorly characterized. The *APOE ɛ4* allele, the most significant common genetic risk factor for dementia,^19^, ^20^, ^21^ exhibits higher penetrance in women,^22^ and may influence the cognitive effects of HRT.^23^, ^24^ Associations between HRT use and dementia could also differ by age at menarche and menopause as these alter the lifetime estrogen exposure in women with evidence that fewer years of exposure is associated with greater neuropathological burden^25^ and dementia risk.^18^, ^26^, ^27^ ^28^ Surgical menopause may further increase risk and alter responsiveness to HRT.^5^, ^29^ Moreover, the “critical window” hypothesis proposes that HRT may be neuroprotective when initiated in the perimenopausal or early postmenopausal period^30^, ^31^ though this has not been systematically investigated.

The aim of the present study was to use data from a large population‐based cohort to identify potential effect modifiers and subgroups that may derive benefit or harm from HRT use.

## 2 METHODS

### 2.1 Study design and participants

We used data from UK Biobank, an ongoing cohort study of more than 500,000 people aged 37 to 73 years at baseline.^32^ Participants were assessed at 22 assessment centers around the United Kingdom between 2006 and 2010.

Analyzes were restricted to postmenopausal women who experienced either natural (spontaneous) or surgical menopause (hysterectomy and/or bilateral oophorectomy). Although a hysterectomy without an oophorectomy does not typically cause an immediate drop in estrogen levels, many women experience lower estrogen levels and earlier menopause likely due to reduced blood flow to the ovaries. Therefore, the groups were combined as a surgical menopause group where early menopause was induced, rather than natural. Age of menopause was self‐reported. For natural menopause it was captured by the question “How old were you when your periods stopped?” (ID 3581). For hysterectomy or oophorectomy, it was captured by the questions “How old were you when you had your hysterectomy?” (ID 2824) / “How old were you when you had BOTH ovaries removed” (ID 3882). Women who had dementia at baseline, were missing information on HRT (whether they ever used HRT or their age at first or last use) or menopausal status were excluded. We followed the Strengthening the Reporting of Observational Studies in Epidemiology (STROBE) guidelines.

### 2.2 Incident dementia

**RESEARCH IN CONTEXT**

1. **Systematic review**: The literature was reviewed using standard sources. Despite biological plausibility, evidence regarding the association between hormone replacement therapy (HRT), which is used by approximately 15% of women aged 45–64 years in England, and cognitive outcomes or dementia risk is conflicting and inconclusive. These discrepancies may reflect the influence of multiple modifying factors, many of which remain poorly characterized.
2. **Interpretation**: This UK Biobank analysis is the most comprehensive prospective analysis to date of the association between HRT and incident dementia in populations subgroups and its potential modulators. We showed that HRT was associated with a 10% reduced dementia risk, and affected by apolipoprotein E (*APOE)* genotype, menopause “type”, lifetime exposure to natural estrogen, and age at initiation of HRT.
3. **Future directions**: UK Biobank contains generic HRT information. Future work should aim to provide nuance by examining the impact of HRT composition (estrogen, progestogen, testosterone), dose, and mode of administration (oral, topical or vaginal) on dementia risk. Furthermore research should explore the mechanisms underpinning the effects of the modulators on HRT‐dementia association to provide further biological plausibility.

Our primary outcome was “all‐cause dementia”, with Alzheimer's disease (AD) and non‐AD dementia (defined as “all‐cause dementia” excluding participants who ever received an AD diagnostic code) as secondary outcomes. The reference group are participants with no dementia diagnosis. The censoring date for time to event analyzes was set to the October 30, 2022 or date of death, whichever was earlier. Details of ascertainment of dementia diagnoses, including the full list of *International Classification of Diseases (ICD)* used are provided in the Supplement.

### 2.3 HRT use

We defined use of HRT, from self‐reported data, as a binary variable, with current users of HRT plus those who had used HRT for at least 1 year as the exposure category, and those who never used HRT or used HRT for less than 12 months as the reference category.

### 2.4 Covariates

We adjusted the model for covariates known to impact on dementia risk and/or HRT use: age; systolic blood pressure; body mass index (BMI); serum cholesterol levels; Townsend Deprivation Index^33^ (all continuous variables); education as five categories; ethnicity as six categories; smoking status categorized as never smoked or smoker; diabetes defined as a binary variable; use of cholesterol lowering medication; use of anti‐hypertensive medication. A specification of the covariates is provided in eTable 1 of the Supplement. In addition, an unadjusted model with no covariates and a partially adjusted model with age, education and smoking status as covariates were trained and results reported in the Supplement.

### 2.5 Statistical analysis

We applied Cox proportional hazard models to estimate the hazard ratios for dementia risk. Multiple stratified models were utilized with HRT use as one category and non‐HRT use as the reference. Confidence intervals are reported at the 95% level and *p*‐values have been reported where appropriate. Cox models were run using Python package *lifelines*.^34^

Given that there are well characterized risk factors for dementia and logical modulators of the HRT effect, we investigated possible modifying effects of the following covariates: *APOE ɛ4* status, surgical menopause, and length of natural estrogen exposure (time between menarche and menopause). Women with missing data on age of menarche or age of menopause were excluded from this sub‐analyzes. *APOE ɛ4* status is available in the genetic data of UK Biobank via two single‐nucleotide polymorphisms: rs429358 and rs7412. A binary variable was derived for APOE status: no ɛ4 alleles or 1–2 ɛ4 alleles. For women who had reproductive surgery, we defined the age of menopause as the earliest age of either surgery.

To explore the window of opportunity hypothesis we estimated the hazard ratios for women starting HRT at different ages compared to the reference category. We considered all women who started HRT earlier/later than a specified age from 40 to 59 years; for example, when considering earlier starting ages of HRT, we took all women who started HRT at ≤ 45years and assessed their risk of dementia compared to women who never took HRT. In addition, we assessed dementia risk for those women who started HRT within time windows of 46–50 years and 51–56 years (inclusive).

## 3 RESULTS

From the original sample of 273,155 women, 89,705 were excluded, as they had not undergone menopause (64,022) or had missing menopause information (12,431), had missing information on HRT (13,154), or because they had dementia at baseline (98), leaving 183,450 included in the analysis (see eFigure 1 in the Supplement). Baseline characteristics are shown in Table 1. During 2,433,320 (mean [SD] follow‐up duration 13.26 [1.96]; median [interquartile range {IQR}] follow‐up of 13.58 [12.83–14.33]) person‐years of follow‐up, there were 3,948 dementia diagnoses, of which 1,993 were AD and 1,955 were either “dementia, unspecified” or a non‐Alzheimer's form of dementia (non‐AD).

**TABLE 1:** Baseline characteristics

Characteristic	Incident dementia (n = 3 948)	No dementia (n = 179 502)	Total (n = 183 450)
Age, mean (SD), y	65.0 (4.1)	60.1 (5.8)	60.3 (5.8)
APOE status			
No E4 Allele, n (%)	1 614 (40.9)	125 664 (70.0)	127 278 (69.4)
1 or 2 E4 Alleles, n (%)	2 167 (54.9)	48 020 (26.8)	50 187 (27.4)
Menopause status^a^, ^b^			
Natural menopause, n (%)	2 782 (70.5)	133 222 (74.2)	136 004 (74.1)
Age at natural menopause, mean (SD), y	50.0 (4.9)	50.3 (4.4)	50.3 (4.4)
Surgical menopause, n (%)	972 (24.6)	40 827 (22.7)	41 799 (22.8)^b^
Age at surgical menopause, mean (SD), y	42.6 (7.6)	42.5 (6.9)	42.5 (6.9)
Natural estrogen exposure^c^			
Natural estrogen exposure, mean (SD), y	35.0 (6.8)	35.5 (6.3)	35.5 (6.3)
Longer natural estrogen, ≥ 37y, n (%)	1 729 (43.8)	87 483 (48.7)	89 212 (48.6)
Shorter natural estrogen, < 37y, n (%)	1 869 (47.3)	81 935 (45.6)	83 804 (45.7)
HRT use^d^			
No HRT use, n (%)	2 177 (55.1)	103 938 (57.9)	106 115 (57.8)
Used HRT, n (%)	1 771 (44.9)	75 564 (42.1)	77 335 (42.2)
HRT duration^e^, mean (SD), y	9.2 (6.1)	7.9 (5.5)	7.9 (5.6)
Education^f^, ^g^			
Other, n (%)	1 349 (34.2)	36 057 (20.1)	37 406 (20.4)
Lower secondary, n (%)	1 480 (37.5)	89 038 (49.6)	90 518 (49.3)
Upper secondary, n (%)	596 (15.1)	41 522 (23.1)	42 118 (23.0)
NVQ, n (%)	354 (9.0)	22 942 (12.8)	23 296 (12.7)
Higher, n (%)	1 326 (33.6)	80 010 (44.6)	81 336 (44.3)
Smoking status^g^			
Never smoked, n (%)	2 135 (54.1)	103 864 (57.9)	105 999 (57.8)
Current or past smoker, n (%)	1 783 (45.2)	75 012 (41.8)	76 795 (41.9)
Ethnicity^g^			
White, n (%)	3 804 (96.4)	171 821 (95.7)	175 625 (95.7)
Asian, n (%)	42 (1.1)	2 537 (1.4)	2 579 (1.4)
Black, n (%)	58 (1.5)	2 365 (1.3)	2 423 (1.3)
Mixed, n (%)	18 (0.5)	854 (0.5)	872 (0.5)
Chinese, n (%)	<5 (0.1)	491 (0.3)	495 (0.3)
Other, n (%)	22 (0.6)	1 434 (0.8)	1456 (0.8)
Systolic blood pressure^g^, mean (SD) (mmHg)	145.5 (20.0)	140.3 (19.6)	140.4 (19.7)
BMI^g^, mean (SD) (kg/m^2^)	27.6 (5.3)	27.2 (5.1)	27.2 (5.1)
Diabetes^g^			
No diabetes, n (%)	3 540 (89.7)	171 817 (95.7)	175 357 (95.6)
Diabetes, n (%)	398 (10.1)	7 328 (4.1)	7 726 (4.2)
Cholesterol^g^ (SD) (mmol/l)	6.0 (1.2)	6.0 (1.1)	6.0 (1.1)
Blood pressure medication^g^			
No blood pressure medication, n (%)	2 522 (63.9)	140 099 (78.0)	142 621 (77.7)
Blood pressure medication, n (%)	1 335 (33.8)	37 489 (20.9)	38 824 (21.2)
Cholesterol drugs^g^			
No cholesterol medication, n (%)	2 668 (67.6)	149 833 (83.5)	152 501 (83.1)
Cholesterol medication, n (%)	1 189 (30.1)	27 755 (15.5)	28 944 (15.8)
Townsend deprivation index^g^, mean (SD)	27.6 (5.3)	27.2 (5.1)	27.2 (5.1)

Abbreviations: APOE, apolipoprotein E; BMI, body mass index; HRT, hormone replacement therapy; NVQ, National Vocational Qualifications; SD, standard deviation;

^a^ Surgical menopause is defined as having had a bilateral oophorectomy or hysterectomy.

^b^ Age at surgical menopause is the age at hysterectomy or age at bilateral oophorectomy. n = 5647 (i.e., 3.1% of the cohort) who had a bilateral oophorectomy or hysterectomy after natural menopause were not included in the analysis.

^c^ Natural estrogen exposure is years between menarche and earliest of natural or surgical menopause.

^d^ No HRT use is defined as never using HRT or using HRT but starting and stopping at the same age. Used HRT is defined as using HRT for at least 1 year or still using HRT at baseline.

^e^ Includes individuals still using HRT.

^f^ Educational categories are specified in the Supplement. Results sum to more than 100% because multiple qualifications could be specified.

^g^ Missing values were imputed using multiple imputations by chained equations with 40 imputations.

### 3.1 Association between HRT and all‐cause dementia

**FIGURE 1:**
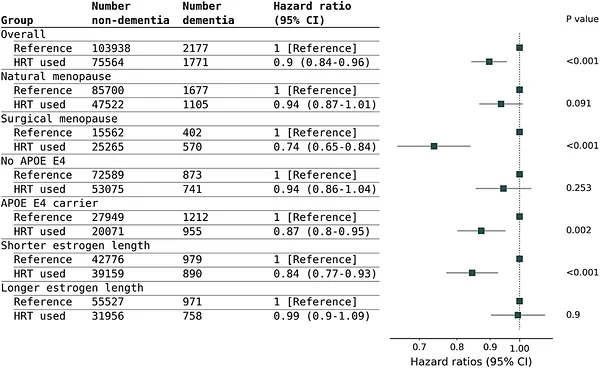
Risk of incident dementia stratified by groups. All results from a cox proportional hazards regression adjusted for: age, education, smoking status, systolic blood pressure, ethnicity, BMI, cholesterol, Townsend Deprivation Index, diabetes, whether taking cholesterol lowering medication, whether taking anti‐hypertensive medications. The reference for each group is those who never took HRT. The women defined as using HRT had used HRT for at least 1 year or were still taking HRT at baseline. Natural menopause is women who reported natural cessation of periods. Surgical menopause is women who had either a hysterectomy and/or bilateral oophorectomy. No APOE ɛ4 women did not have any ɛ4 alleles. APOE ɛ4 carriers had either one or two ɛ4 alleles. Estrogen length is defined as time between menarche and the earliest of age at menopause, age at hysterectomy or bilateral oophorectomy. Shorter estrogen length is less than 37 years of natural estrogen length. Longer estrogen length is 37 years or more of natural estrogen length. APOE, apolipoprotein E; BMI, body mass index; HRT, hormone replacement therapy.

HRT was associated with a reduced risk of dementia with a hazard ratio (HR) of 0.90 (95% confidence interval [CI], 0.84–0.96; *p =* 0.001) (Figure 1), in a Cox's proportional hazards regression model adjusted for age, education, smoking status, systolic blood pressure, ethnicity, BMI, cholesterol, Townsend deprivation index, diabetes, and use of cholesterol lowering or anti‐hypertensive medications. For women who experienced natural menopause, this association was not statistically significant with a HR of 0.94 (95% CI, 0.87–1.01; *p =* 0.091). For women who underwent surgical menopause HRT was associated with a reduction in dementia risk with a HR of 0.74 (95% CI, 0.65–0.84; *p < *0.001). Although no overall significant interaction between *APOE* and HRT was evident (eFigure 2), the association of HRT with dementia was only significant for those with at least one allele (HR, 0.87; 95% CI, 0.80–0.95; *p =* 0.002, Figure 1). Significant interaction terms were evident between both menopause type (*p* = 0.001; eFigure 3) and length of estrogen exposure (*p* = 0.018; eFigure 4) and HRT, with associations between HRT and dementia evident in women who had undergone surgical menopause (HR, 0.74; 95% CI, 0.65–0.84; Figure 1) or exposed to natural estrogen for less than 37 years (median value between menarche and either natural menopause or surgical menopause) (HR, 0.84; 95% CI, 0.77–0.93; Figure 1).

### 3.2 Combinations of menopause type, APOE ɛ4 status, and length of exposure to natural estrogen

The association between HRT and dementia depends on the combined effects of menopause type (natural or surgical menopause), *APOE ɛ4* status, and length of exposure to natural estrogen (eFigure 5 and 6). For women who had a natural menopause, HRT is not associated with dementia risk. The HR for women with natural menopause who are *APOE ɛ4* and had a shorter natural estrogen exposure was 0.86 (95% CI, 0.73–1.03; *p* = 0.10). For women who had surgical menopause, associations were evident in both genotype groups (HR 0.72; 95% CI, 0.60–0.86; *p* < 0.001 in *APOE ɛ4* versus HR 0.80; 95% CI, 0.65–0.97; *p* = 0.03 in non‐ *APOE ɛ4*) (eFigure 5).

### 3.3 Association of HRT and AD

**FIGURE 2:**
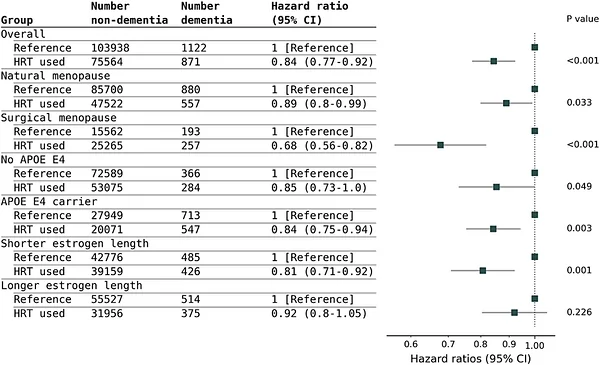
Risk of incident AD stratified by groups. All results from a Cox proportional hazards regression adjusted for: age, education, smoking status, systolic blood pressure, ethnicity, BMI, cholesterol, Townsend deprivation index, diabetes, whether taking cholesterol lowering medication, whether taking anti‐hypertensive medications. The reference for each group is those who never took HRT or took HRT but started and stopped at the same age. The HRT used women used HRT for at least 1 year or were still taking HRT at baseline. Natural menopause is women who reported cessation of periods and who had not had a bilateral oophorectomy or a hysterectomy. Surgical menopause are women who had either a hysterectomy and/or bilateral oophorectomy. No APOE ɛ4 women did not have any ɛ4 alleles. APOE ɛ4 carriers had either one or two ɛ4 alleles. Estrogen length is defined as time between menarche and the earliest of age at menopause, age at hysterectomy, or age at bilateral oophorectomy. Shorter estrogen length is less than 37 years of estrogen length. Longer estrogen length is 37 years or more of estrogen length. APOE, apolipoprotein E; AD, Alzheimer's disease; BMI, body mass index; HRT, hormone replacement therapy.

HRT was associated with reduced AD risk with a HR of 0.84 (95% CI, 0.77–0.92; *p* < 0.001) (Figure 2). For women who had a natural menopause, the HR was 0.89 (95% CI, 0.80–0.99; *p* = 0.03), and for those who had surgical menopause, the HR was 0.68 (95% CI, 0.56–0.82; *p* < 0.001). The HR was similar for women with no *APOE ɛ4* allele or with at least one at 0.85 (95% CI, 0.73–1.00; *p* = 0.05) and 0.84 (95% CI, 0.75–0.94; *p* = 0.003), respectively. The HR for women exposed to fewer than 37 years of natural estrogen was 0.81 (95% CI, 0.71–0.92; *p* = 0.001), while for those exposed to 37 or more years of natural estrogen the HR was 0.92 (95% CI, 0.80–1.05; *p* = 0.23).

### 3.4 Association of HRT and non‐AD

**FIGURE 3:**
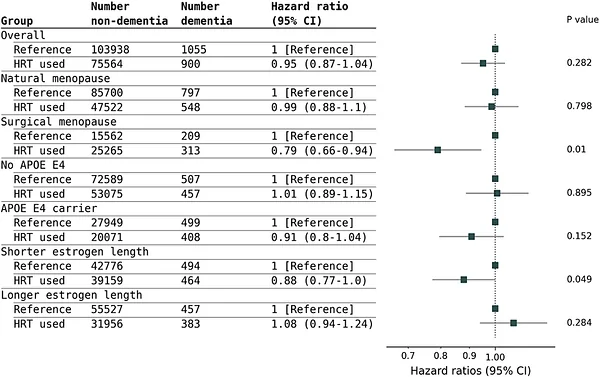
Risk of incident non‐AD stratified by groups. All results from a Cox proportional hazards regression adjusted for: age, education, smoking status, systolic blood pressure, ethnicity, BMI, cholesterol, Townsend deprivation index, diabetes, whether taking cholesterol lowering medication, whether taking anti‐hypertensive medications. The reference for each group is those who never took HRT or took HRT but started and stopped at the same age. The HRT used women used HRT for at least 1 year or were still taking HRT at baseline. Natural menopause is women who reported cessation of periods and who had not had a bilateral oophorectomy or a hysterectomy. Surgical menopause are women who had either a hysterectomy and/or bilateral oophorectomy. No APOE ɛ4 women did not have any ɛ4 alleles. APOE ɛ4 carriers had either one or two ɛ4 alleles. Estrogen length is defined as time between menarche and the earliest of age at menopause, age at hysterectomy, or age at bilateral oophorectomy. Shorter estrogen length is less than 37 years of estrogen length. Longer estrogen length is 37 years or more of estrogen length. AD, Alzheimer's disease; APOE, apolipoprotein E; BMI, body mass index; HRT, hormone replacement therapy.

There was no significant HRT association for non‐AD for all women, with a HR of 0.95 (95% CI, 0.87–1.04; *p* = 0.28) (Figure 3) or for those who had a natural menopause with a HR of 0.99 (95% CI, 0.88–1.10; *p* = 0.80). For those women who had surgical menopause the HR was 0.79 (95% CI, 0.66–0.94; *p* = 0.01). The HRT association was not significant when stratifying into those with or without APOE ɛ4 alleles. For those women with fewer than 37 years of exposure to natural estrogen there was an association between HRT use and non‐AD with a HR of 0.88 (95% CI, 0.77–1.00; *p* = 0.049) while for those with 37 or more years of exposure to natural estrogen the HR was not significant.

### 3.5 Association between age at first use of HRT and dementia risk

**FIGURE 4:**
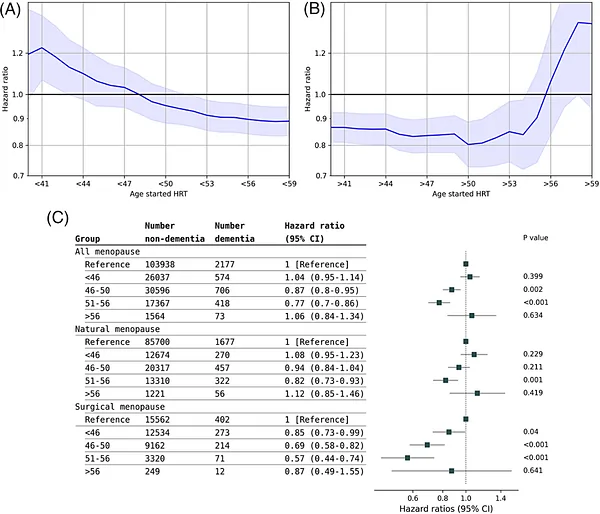
Risk of incident dementia by starting age of HRT. (A) Hazard ratio for those that started using HRT before the stated age with the reference those women who never used HRT. Solid line is the hazard ratio and the shaded area is the 95% confidence intervals. (B) Hazard ratio for those that started using HRT after the stated age with the reference those women who never used HRT. Solid line is the hazard ratio and the shaded area is the 95% confidence intervals. (C) The starting age of HRT is split into four groups: those who started before 46 years, between 46 and 50 years (inclusive), between 51 and 56 years (inclusive), and those who started after 56 years. As well as all the women who had had menopause (All menopause) there are also stratifications into those who had a natural menopause or a surgical menopause (bilateral oophorectomy and/or hysterectomy). HRT, hormone replacement therapy.

The association between HRT and dementia risk was dependent on the age of starting HRT (Figure 4). For all postmenopausal women starting HRT between 46 and 50 years (inclusive) or 51 and 56 years (inclusive) was associated with reduced dementia risk with HRs of 0.87 (95% CI, 0.80–0.95; *p* = 0.002) and 0.77 (95% CI, 0.70–0.86; *p* < 0.001), respectively. For women who had a natural menopause starting HRT between 51 and 56 years was associated with reduced dementia risk with a HR of 0.82 (95% CI, 0.73–0.93; *p* = 0.001) but starting HRT between 46 and 50 years was not significantly associated with reduced risk. Starting HRT either earlier than 47 years or later than 56 years was associated with a non‐significant increased dementia risk. For women who had surgical menopause, HRT was associated with reduced dementia risk when started < 46y, between 46 and 50 years and 51 and 56 years with HRs of 0.85 (95% CI, 0.73‐0.99: *p* < 0.04), 0.69 (95% CI, 0.58–0.82; *p* < 0.001) and 0.57 (95% CI, 0.44–0.74; *p* < 0.001), respectively. An additional description of Figure 4 is available in the Supplement.

### 3.6 Sensitivity analyses

There were 9,065 women who used HRT for less than 1 year (starting age and age at last use are the same) who are included in the reference (non‐HRT use) group. If these women are excluded from the analysis the results do not change significantly (eFigure 7).

The proportion of women using HRT is higher in older women; in addition, younger women at the baseline assessment who were not HRT users may start using HRT when they are older. Removing younger women from the analysis reduces these two effects (see Supplement for additional discussion) and the overall HRT association remains similar (eFigure 8).

Women who are diagnosed with dementia soon after baseline may have already had cognitive impairment which may affect the quality of the data provided; we removed women diagnosed with dementia within 2, 4, 6, and 8 years after baseline assessment (eFigure 9) with no significant change in the HRT effect for any of these analyzes.

The impact of surgery type and early versus late menarche and menopause are presented in eFigures 10–13 in the Supplement. We defined *APOE ɛ4* carriers as those with one or two ɛ4 alleles, in eFigure 14 of the Supplement we show hazard ratios for separated carriers of the *APOE ɛ2, ɛ3* and *ɛ4* alleles. In the main analysis, *APOE ɛ2ɛ4* is included within the *APOE ɛ4* carrier group. As the *APOE ɛ2* allele is associated with reduced dementia risk, we reanalyzed dementia risk with HRT with the *ɛ2ɛ4* carriers removed (eFigure 15). There were no significant changes in results.

The analysis of effect of age of starting HRT was repeated for AD and non‐AD with results in eFigure 16 and 17 of the Supplement. Results for starting HRT stratified by women who had a natural menopause and surgical menopause are in eFigures 18 and 19 of the Supplement.

The main analyses were repeated with two different sets of covariates: no covariates; and only age, education, and smoking status as covariates. These results are reported in eFigures 20–27 in the Supplement. The pattern of the results was similar to the fully adjusted models used in the primary analyses.

## 4 DISCUSSION

This UK Biobank analysis is the most comprehensive prospective analysis to date of the association between HRT and incident dementia and its potential mediators. We showed that HRT was associated with a 10% reduced dementia risk, and differed by *APOE* genotype, menopause “type” and lifetime exposure to natural estrogen. Significant associations between HRT and dementia were only evident in the women who had initiated HRT between 46 and 56 years supporting the “window of opportunity” hypothesis for HRT benefits. However, for older ages we cannot preclude the possibility that reverse causation or confounding by indication affected the associations.

Early observation studies reported that estrogen therapy was associated with reduced dementia.^35^ However, the only RCT with incident dementia conducted to date, the WHIMS, observed an increased incidence of dementia over 5 years following estrogen plus progestogen HRT use in participants > 65 years.^10^ Subsequent RCTS in younger participants, but nevertheless mainly in early postmenopause, have observed little effect of HRT on cognitive outcomes.^36^, ^37^ Our finding provide nuance to the importance of HRT timing, supporting the healthy cell bias hypothesis,^38^ of greater benefit in the absence of significant existing neuropathology if HRT is taken between 46 and 56 years which typically represent the perimenopausal and early postmenopausal periods. Mechanistic analyses suggest that the shorter the delay between menopause and onset of treatment, the higher chance of favorable effects of HRT on the brain.^2^, ^39^ HRT timing has also been identified as important for cardiovascular benefit.^40^

The basis for our observation of stronger associations and potential benefit of HRT in surgical versus natural menopause needs consideration as it may in part reflect the HRT formulation. In natural menopause a combined estrogen–progestogen HRT is now typically prescribed with progestogen preventing endothelial hyperplasia in women with a functional uterus. In surgical menopause induced by a bilateral oophorectomy, a hysterectomy is common and estrogen only HRT formulations are typically subsequently prescribed. The WHIMS and large cohort studies have consistently reported that estrogen only therapy is associated with lower dementia risk compared to the combined HRT.^9^, ^10^, ^41^ Lack of information on HRT formulation is a limitation of the current analysis.

We observed stronger associations between HRT use and dementia risk in carriers of the *APOE ɛ4* allele relative to non‐carriers, which is consistent with our previous smaller scale cross‐sectional analysis in the EPAD cohort, where the association between HRT and cognition and brain volumes in the media temporal lobe were only evident in *APOE ɛ4* ^24^. The *APOE ɛ4* allele is associated with accelerated neurocognitive decline and higher dementia risk, with an allele frequency of 20–25% in the general population versus 50–75% in AD.^42^ Evidence of greater benefit in *APOE ɛ4* is potentially due to the overlapping molecular targets of APOE and estrogen, with the *APOE ɛ4* allele negatively impacting synaptic plasticity, BBB function, glucose metabolism and neuroinflammation, relative to the wildtype genotype.^43^ However, these stronger associations in *APOE ɛ4* should be interpreted with a degree of caution as no significant overall *APOE ɛ4* allele*HRT interaction was observed.

### 4.1 Strengths and limitations

A strength of our study is that the amount of available data, alongside the follow‐up for dementia diagnosis, gives us a large dataset to assess the relationship between HRT and dementia risk, with almost 4000 incident cases over up to 16 years follow‐up. We were able to stratify women into various subgroups to show that the association between HRT and dementia risk varies considerably. The availability of a significant amount of additional data in UK Biobank allowed a comprehensive sensitivity analysis to multiple potential factors and confounders.

A limitation of our study is that results are associations and not causal. HRT use is subject to selection (healthy‐user) bias, as women who use HRT can differ systematically from non‐users in characteristics that are also associated with dementia risk, including socioeconomic status, educational attainment, ethnicity, and lifestyle factors. Although these factors were in large part corrected for by their addition as covariates to the model, we cannot preclude the possibility that residual confounding in part explained the modest 10% reduction in dementia risk in the group as a whole associated with HRT use. As mentioned above the lack of information on HRT formulation (estrogen only or estrogen plus progestogen), dose, and mode of administration (topical, oral, vaginal) in UK Biobank prevents any analysis on the impact of these important variables. UK Biobank predominately includes women of European descent, with participants disproportionally drawn for more socioeconomically advantaged groups.^44^ In addition, although UK Biobank is a large study we lack statistical power to draw strong conclusions when we stratify by groups or dementia sub‐types. Diagnoses are primarily from secondary care (hospital inpatient admissions), which can affect dementia classification and often does not capture milder cases of dementia. Future work integrating more primary care records and specialized dementia diagnosis would strengthen the analysis.

We have demonstrated associations between HRT use and age at initiation with dementia risk; an important area for future research is to determine how duration of HRT use influences these associations.

Conclusion: In UK Biobank participants, HRT was associated with reduced dementia risk with larger associations for AD than non‐AD. The associations are greatest in those who had undergone surgical menopause, and when HRT is initiated between ages 46 and 56 years. The findings suggest a potential differential response to HRT in female subgroups and rationalize the need for confirmation in an RCT setting to inform a more personalised approach to HRT prescribing for reducing population dementia risk.

## CONFLICT OF INTEREST STATEMENT

The authors declare no conflicts of interest. Author disclosures are available in the Supporting Information.

## CONSENT STATEMENT

All participants provided informed consent to the UK Biobank Access Procedures and agreed to the use of their anonymized data for health‐related research purposes. For the specific study, we acknowledge that participants' data was used in line with the UK Biobank ethical approval, which included their initial consent.

## Supporting information



Supporting Information

Supporting Information
