# Supplementary material for: Hormone replacement therapy and dementia risk among postmenopausal women: Identifying responsive subgroups in the UK Biobank
Source: Alzheimers Dement. 2026 Aug 26;22(8):e71679. doi: 10.1002/alz.71679 (PMC13508136; doi:10.1002/alz.71679)
Supplement: Supplementary file 2 [file ALZ-22-e71679-s002.docx]

eMethods

eFigure 1 Flowchart of included participants in the overall analysis

eTable 1 Covariates used in the models

*eFigure 2* *A decision tree with hazard ratios for the association between HRT use and dementia with different stratifications*

*eFigure 3* *Further decision trees with hazard ratio for the association between HRT use and dementia with different stratifications*

eFigure 4 Risk of incident dementia removing women with short term HRT use

*eFigure 5 Risk of incident dementia removing younger women from the analysis*

eFigure 6 Risk of incident dementia removing women diagnosed with dementia within stated number of years of baseline

eFigure 7 Risk of incident dementia stratified by surgery subtypes

eFigure 8 Risk of incident dementia stratified by Early/Late Menarche/Menopause for all-menopause

eFigure 9 Risk of incident dementia stratified by Early/Late Menarche/Menopause for natural-menopause

eFigure 10 Risk of incident dementia stratified by Early/Late Menarche/Menopause for surgical-menopause

eFigure 11 Risk of incident dementia for different combinations of APOE

eFigure 12 Risk of incident dementia excluding APOE ɛ2ɛ4 carriers

eFigure 13 HRT-APOE interaction for risk of incident dementia

*eFigure 14 HRT-length of exposure to natural estrogen interaction for risk of incident dementia*

*eFigure 15 HRT- surgery interaction for risk of incident dementia*

eFigure 16 Risk of incident AD by starting age of HRT

eFigure 17 Risk of incident non-AD by starting age of HRT

eFigure 18 Risk of incident dementia by starting age of HRT for women who had a natural menopause

eFigure 19 Risk of incident dementia by starting age of HRT for women who had a surgical menopause

eFigure 20 Risk of incident dementia stratified by groups

eFigure 21 Risk of incident AD stratified by groups

eFigure 22 Risk of incident non-AD stratified by groups

eFigure 23 Risk of incident dementia by starting age of HRT

eFigure 24 Risk of incident dementia stratified by groups

eFigure 25 Risk of incident AD stratified by groups

eFigure 26 Risk of incident non-AD stratified by groups

eFigure 27 Risk of incident dementia by starting age of HRT

**eMethods**

Included participants

eFigure 1 Flowchart of included participants in the overall analysis

Study design and participants

UK Biobank has ethical approval from the North West Multi-Centre Research Ethics Committee (reference 11/NW/0382) and all participants provided informed consent through electronic signature at baseline assessment. Because data were deidentified, this study did not require additional approval. Access to UK Biobank was granted under Application Number 14631.

Incident dementia ascertainment

We ascertained dementia diagnoses from three sources of linked medical records data: 1) hospital inpatient records containing data on primary and secondary diagnoses obtained from the Hospital Episode Statistics (HES) for England, Scottish Morbidity Record data for Scotland, and the Patient Episode Database for Wales; 2) death register data on underlying/contributory cause of death provided by the National Health Service Digital for England and Wales and the Information and Statistics Division for Scotland; and 3) primary care (General Practice, GP) diagnosis data were available in 45% of the cohort, up to 2016 or 2017 depending on data provider (<https://biobank.ndph.ox.ac.uk/ukb/label.cgi?id=3000>). HES and death record diagnoses were recorded using the *International Classification of Diseases* (*ICD*) coding system, from: Casanova et al. (2024) "Iron and risk of dementia: Mendelian randomisation analysis in UK Biobank" J Med Genetic. <https://doi.org/10.1136/jmg-2023-109295>.

condition  vocabulary  code
 dementia  ICD10  F00
 dementia  ICD10  F01
 dementia  ICD10  F02
 dementia  ICD10  F03
 dementia  ICD10  G30
 dementia  ICD10  A81.0
 dementia  ICD10  F05.1
 dementia  ICD10  F10.6
 dementia  ICD10  G31.0
 dementia  ICD10  G31.1
 dementia  ICD10  G31.8
 dementia  ICD10  I67.3
 dementia  Read2  A410.
 dementia  Read2  A411.
 dementia  Read2  A4110
 dementia  Read2  A4110
 dementia  Read2  E00..
 dementia  Read2  E00..
 dementia  Read2  E00..
 dementia  Read2  E000.
 dementia  Read2  E001.
 dementia  Read2  E0010
 dementia  Read2  E0011
 dementia  Read2  E0012
 dementia  Read2  E0013
 dementia  Read2  E001z
 dementia  Read2  E002.
 dementia  Read2  E0020
 dementia  Read2  E0021
 dementia  Read2  E002z
 dementia  Read2  E003.
 dementia  Read2  E004.
 dementia  Read2  E004.
 dementia  Read2  E0040
 dementia  Read2  E0041
 dementia  Read2  E0042
 dementia  Read2  E0043
 dementia  Read2  E004z
 dementia  Read2  E00y.
 dementia  Read2  E041.
 dementia  Read2  Eu00.
 dementia  Read2  Eu000
 dementia  Read2  Eu000
 dementia  Read2  Eu000
 dementia  Read2  Eu000
 dementia  Read2  Eu001
 dementia  Read2  Eu001
 dementia  Read2  Eu001
 dementia  Read2  Eu001
 dementia  Read2  Eu002
 dementia  Read2  Eu00z
 dementia  Read2  Eu00z
 dementia  Read2  Eu01.
 dementia  Read2  Eu01.
 dementia  Read2  Eu010
 dementia  Read2  Eu011
 dementia  Read2  Eu011
 dementia  Read2  Eu012
 dementia  Read2  Eu013
 dementia  Read2  Eu01y
 dementia  Read2  Eu01z
 dementia  Read2  Eu02.
 dementia  Read2  Eu020
 dementia  Read2  Eu021
 dementia  Read2  Eu022
 dementia  Read2  Eu023
 dementia  Read2  Eu025
 dementia  Read2  Eu02y
 dementia  Read2  Eu02z
 dementia  Read2  Eu02z
 dementia  Read2  Eu02z
 dementia  Read2  Eu02z
 dementia  Read2  Eu02z
 dementia  Read2  Eu02z
 dementia  Read2  Eu02z
 dementia  Read2  F110.
 dementia  Read2  F1100
 dementia  Read2  F1101
 dementia  Read2  F111.
 dementia  Read2  F112.
 dementia  Read2  F116.
 dementia  Read2  F118.
 dementia  Read2  F11x7
 dementia  Read2  F21y2
 dementia  Read2  F21y2
 dementia  Read2  Fyu30
 dementia  CTV3  A410.
 dementia  CTV3  A410.
 dementia  CTV3  A411.
 dementia  CTV3  A411.
 dementia  CTV3  A411.
 dementia  CTV3  A411.
 dementia  CTV3  E000.
 dementia  CTV3  E001.
 dementia  CTV3  E0010
 dementia  CTV3  E0011
 dementia  CTV3  E0012
 dementia  CTV3  E0013
 dementia  CTV3  E001z
 dementia  CTV3  E002.
 dementia  CTV3  E0020
 dementia  CTV3  E0021
 dementia  CTV3  E002z
 dementia  CTV3  E003.
 dementia  CTV3  E004.
 dementia  CTV3  E004.
 dementia  CTV3  E004.
 dementia  CTV3  E0040
 dementia  CTV3  E0041
 dementia  CTV3  E0042
 dementia  CTV3  E0043
 dementia  CTV3  E004z
 dementia  CTV3  E00z.
 dementia  CTV3  E00z.
 dementia  CTV3  E041.
 dementia  CTV3  Eu00.
 dementia  CTV3  Eu00.
 dementia  CTV3  Eu00.
 dementia  CTV3  Eu002
 dementia  CTV3  Eu00z
 dementia  CTV3  Eu011
 dementia  CTV3  Eu011
 dementia  CTV3  Eu011
 dementia  CTV3  Eu01y
 dementia  CTV3  Eu01z
 dementia  CTV3  Eu02.
 dementia  CTV3  Eu020
 dementia  CTV3  Eu021
 dementia  CTV3  Eu022
 dementia  CTV3  Eu023
 dementia  CTV3  Eu02y
 dementia  CTV3  Eu041
 dementia  CTV3  F110.
 dementia  CTV3  F110.
 dementia  CTV3  F111.
 dementia  CTV3  F112.
 dementia  CTV3  F11x7
 dementia  CTV3  F21y2
 dementia  CTV3  F21y2
 dementia  CTV3  F21y2
 dementia  CTV3  F21y2
 dementia  CTV3  F21y2
 dementia  CTV3  Fyu30
 dementia  CTV3  X002w
 dementia  CTV3  X002x
 dementia  CTV3  X002x
 dementia  CTV3  X002x
 dementia  CTV3  X002y
 dementia  CTV3  X002z
 dementia  CTV3  X0030
 dementia  CTV3  X0030
 dementia  CTV3  X0030
 dementia  CTV3  X0031
 dementia  CTV3  X0032
 dementia  CTV3  X0033
 dementia  CTV3  X0034
 dementia  CTV3  X0035
 dementia  CTV3  X0036
 dementia  CTV3  X0037
 dementia  CTV3  X0037
 dementia  CTV3  X0039
 dementia  CTV3  X003A
 dementia  CTV3  X003A
 dementia  CTV3  X003A
 dementia  CTV3  X003A
 dementia  CTV3  X003A
 dementia  CTV3  X003A
 dementia  CTV3  X003A
 dementia  CTV3  X003A
 dementia  CTV3  X003G
 dementia  CTV3  X003H
 dementia  CTV3  X003I
 dementia  CTV3  X003J
 dementia  CTV3  X003J
 dementia  CTV3  X003R
 dementia  CTV3  X003T
 dementia  CTV3  X003V
 dementia  CTV3  X003W
 dementia  CTV3  X003X
 dementia  CTV3  X003Y
 dementia  CTV3  X00R2
 dementia  CTV3  X00R2
 dementia  CTV3  XE1aG
 dementia  CTV3  XE1aG
 dementia  CTV3  XE1aG
 dementia  CTV3  XE1aG
 dementia  CTV3  XE1aG
 dementia  CTV3  XE1Xs
 dementia  CTV3  XE1Xs
 dementia  CTV3  XE1Xs
 dementia  CTV3  XE1Xt
 dementia  CTV3  XE1Z6
 dementia  CTV3  Y00dJ
 dementia  CTV3  Y00dM
 dementia  CTV3  Y00Gv
 dementia  CTV3  Y00Gw
 dementia  CTV3  Y00H0
 dementia  CTV3  Y00H2
 dementia  CTV3  Y00H5
 dementia  CTV3  Y00H7
 dementia  CTV3  Y00HC
 dementia  CTV3  Y00Hk
 dementia  CTV3  Y00HL
 dementia  CTV3  Y00Hv
 dementia  CTV3  Y00Hw
 dementia  CTV3  Y00Hx
 dementia  CTV3  Y00Hz
 dementia  CTV3  Y00I0
 dementia  CTV3  Y00I1
 dementia  CTV3  Y00La
 dementia  CTV3  Y016a
 dementia  CTV3  Y016b
 dementia  CTV3  Y016c
 dementia  CTV3  Y016d
 dementia  CTV3  Y016e
 dementia  CTV3  Y016f
 dementia  CTV3  Y016M
 dementia  CTV3  Y016N
 dementia  CTV3  Y016O
 dementia  CTV3  Y016P
 dementia  CTV3  Y016S
 dementia  CTV3  Y016T
 dementia  CTV3  Y016U
 dementia  CTV3  Y016V
 dementia  CTV3  Y016w
 dementia  CTV3  Y016W
 dementia  CTV3  Y016x
 dementia  CTV3  Y016X
 dementia  CTV3  Y016y
 dementia  CTV3  Y016Y
 dementia  CTV3  Y016z
 dementia  CTV3  Y016Z
 dementia  CTV3  Y0170
 dementia  CTV3  Ya1Sy
 dementia  CTV3  Ya2nk
 dementia  CTV3  YadT0
 dementia  CTV3  Yady4
 dementia  CTV3  Yao4I
 dementia  CTV3  YaxJd
 dementia  CTV3  YaxJe
 dementia  CTV3  YMAhP
 dementia  CTV3  YMAhQ
 dementia  CTV3  YMB0i
 dementia  CTV3  YMB0l
 dementia  CTV3  YMB0m
 dementia  CTV3  YMB0s
 dementia  CTV3  YMB0t
 dementia  CTV3  YMB0u
 dementia  CTV3  YMB0v
 dementia  CTV3  YMB0w
 dementia  CTV3  YMB0x
 dementia  CTV3  YMB0y
 dementia  CTV3  YMB10
 dementia  CTV3  YMB11
 dementia  CTV3  YMB15
 dementia  CTV3  YMB1Z
 dementia  CTV3  YMBwl
 dementia  CTV3  YMJwc
 dementia  CTV3  YMK1N
 dementia  CTV3  YMK1O
 dementia  CTV3  YMK1P
 dementia_ad  ICD10  F00
 dementia_ad  ICD10  G30
 dementia_ad  Read2  Eu00.
 dementia_ad  Read2  Eu000
 dementia_ad  Read2  Eu000
 dementia_ad  Read2  Eu000
 dementia_ad  Read2  Eu000
 dementia_ad  Read2  Eu001
 dementia_ad  Read2  Eu001
 dementia_ad  Read2  Eu001
 dementia_ad  Read2  Eu001
 dementia_ad  Read2  Eu002
 dementia_ad  Read2  Eu00z
 dementia_ad  Read2  Eu00z
 dementia_ad  Read2  F110.
 dementia_ad  Read2  F1100
 dementia_ad  Read2  F1101
 dementia_ad  Read2  Fyu30
 dementia_ad  CTV3  Eu00.
 dementia_ad  CTV3  Eu00.
 dementia_ad  CTV3  Eu00.
 dementia_ad  CTV3  Eu002
 dementia_ad  CTV3  Eu00z
 dementia_ad  CTV3  F110.
 dementia_ad  CTV3  F110.
 dementia_ad  CTV3  Fyu30
 dementia_ad  CTV3  X002x
 dementia_ad  CTV3  X002x
 dementia_ad  CTV3  X002x
 dementia_ad  CTV3  X002y
 dementia_ad  CTV3  X002z
 dementia_ad  CTV3  X0030
 dementia_ad  CTV3  X0030
 dementia_ad  CTV3  X0030
 dementia_ad  CTV3  X0031
 dementia_ad  CTV3  X0032
 dementia_ad  CTV3  X0033
 dementia_ad  CTV3  X003G
 dementia_ad  CTV3  Y00Gw
 dementia_ad  CTV3  Y00H0
 dementia_ad  CTV3  Y00H2
 dementia_ad  CTV3  Y00H5
 dementia_ad  CTV3  Y00H7
 dementia_ad  CTV3  YMAhP
 dementia_ad  CTV3  YMAhQ
 dementia_ad  CTV3  YMB0i
 dementia_ad  CTV3  YMB0l
 dementia_ad  CTV3  YMB0m
 dementia_vasc  ICD10  F01
 dementia_vasc  Read2  E004.
 dementia_vasc  Read2  E004.
 dementia_vasc  Read2  E0040
 dementia_vasc  Read2  E0041
 dementia_vasc  Read2  E0042
 dementia_vasc  Read2  E0043
 dementia_vasc  Read2  E004z
 dementia_vasc  Read2  Eu01.
 dementia_vasc  Read2  Eu01.
 dementia_vasc  Read2  Eu010
 dementia_vasc  Read2  Eu011
 dementia_vasc  Read2  Eu012
 dementia_vasc  Read2  Eu013
 dementia_vasc  Read2  Eu01y
 dementia_vasc  Read2  Eu01z
 dementia_vasc  Read2  F21y2
 dementia_vasc  Read2  F21y2
 dementia_vasc  CTV3  E004.
 dementia_vasc  CTV3  E004.
 dementia_vasc  CTV3  E004.
 dementia_vasc  CTV3  E0040
 dementia_vasc  CTV3  E0041
 dementia_vasc  CTV3  E0042
 dementia_vasc  CTV3  E0043
 dementia_vasc  CTV3  E004z
 dementia_vasc  CTV3  Eu011
 dementia_vasc  CTV3  Eu011
 dementia_vasc  CTV3  Eu011
 dementia_vasc  CTV3  Eu01y
 dementia_vasc  CTV3  Eu01z
 dementia_vasc  CTV3  F21y2
 dementia_vasc  CTV3  F21y2
 dementia_vasc  CTV3  F21y2
 dementia_vasc  CTV3  F21y2
 dementia_vasc  CTV3  F21y2
 dementia_vasc  CTV3  X003R
 dementia_vasc  CTV3  X003T
 dementia_vasc  CTV3  X003V
 dementia_vasc  CTV3  XE1Xs
 dementia_vasc  CTV3  XE1Xs
 dementia_vasc  CTV3  XE1Xs
 dementia_vasc  CTV3  Y00dJ
 dementia_vasc  CTV3  Y00dM
 dementia_vasc  CTV3  Y00Hv
 dementia_vasc  CTV3  Y00Hw
 dementia_vasc  CTV3  Y00Hx
 dementia_vasc  CTV3  Y00Hz
 dementia_vasc  CTV3  Y00I0
 dementia_vasc  CTV3  Y00I1
 dementia_vasc  CTV3  Y016w
 dementia_vasc  CTV3  Y016x
 dementia_vasc  CTV3  Y016y
 dementia_vasc  CTV3  Y016z
 dementia_vasc  CTV3  Y0170
 dementia_vasc  CTV3  YadT0
 dementia_vasc  CTV3  YMB0s
 dementia_vasc  CTV3  YMB0t

GP data are recorded as Read2 or CTV3 codes, depending on the data provider.

HRT definition

The exposure of interest was use of HRT as defined by UKBB fields at baseline: ever used HRT (UKBB ID 2814), age started HRT (UKBB ID 3536) and age last used HRT (UKBB ID 3546). We define no HRT use as those women saying they never used HRT or saying they did use HRT but then stating the start and last use age as the same (i.e. started and stopped HRT at the same age). We excluded from all analysis women who did not know if they used HRT or refused to answer, and also if they said they did use HRT but did not answer the age they started or last used HRT.

Proportional hazards assumption

The proportional hazards assumptions were tested and holds for all the exposures and variables stratified on and for most of the covariates. The proportional hazards assumptions were only violated for the age variable (as a covariate) - but we tested stratifications by groups of women by age as well (see eFigure 5) to check this effect – the results remain consistent when introducing an age cutoff implying no effect from this violation of proportional hazards.

Additional stratifications by APOE4 status, age of menarche, age at menopause, length of oestrogen and reproductive surgery

We stratified the results by APOE ɛ4 status: we defined a APOE ɛ4 carrier as having one or two APOE ɛ4 alleles. APOE alleles are denoted as 0 or 1 with 0: APOE ɛ3/3, APOE ɛ2/2, APOE ɛ2/3 and 1: APOE ɛ3/4, APOE ɛ2/4, APOE ɛ4/4. We also performed a sensitivity analysis removing the APOE ɛ2/4 carriers entirely.

Women with natural menopause were those who answered their periods had stopped and had never had a hysterectomy or a bilateral oophorectomy. We defined women with surgical menopause as those who had one (or both) of a bilateral oophorectomy or a hysterectomy; for the surgical menopause any woman whose periods stopped before they had surgery were excluded from the surgical menopause-natural menopause sub-analyses.

We additionally stratified by age at menarche (UKBB ID 2714), age at menopause and years between menarche and menopause. Age of spontaneous (natural) menopause is given by UKBB ID 3581. For women who had either a hysterectomy and/or bilateral oophorectomy (given by UKBB IDs 2724, 3591, 2834) we defined the age of menopause as the earliest age of surgery or stated menopause age (3581). We stratified into pairs of groups for age of menarche (14 or younger, 15 or older), age of menopause (44 or younger, 45 or older) and length of natural estrogen (36 years or fewer, 37 years or more). Any that have missing data on age of menarche or age of menopause were removed from that stratification.

We also stratified by age of starting HRT to investigate a possible window of opportunity. The reference class is the same as for the other analyses: women who never took HRT or started and stopped at the same age. We compare the reference class with women who started HRT between the ages of 46-50 years (inclusive) and (separately) 51-56 years (inclusive). We also explore the hazard ratio changes if we compare all women who started HRT before a set of ages between 41 and 59 years. Separately we do the same but for all women who started HRT after that age. For these analyses we additionally stratified into women who had any menopause, a natural menopause, or a surgical menopause.

Covariates

In eTable 1 lists the covariates used in the adjusted model, an additional column specifies which variables were used in the partially adjusted model.

eTable 1 Covariates used in the models

| Covariate | Model | ID | Details |
| --- | --- | --- | --- |
| Age | 2,3 | 21022 | Continuous measure, years |
| Education | 2,3 | 6138 | Five categories: none of the above; CSEs or equivalent & O levels/GCSEs or equivalent; A levels/AS levels or equivalent; NVQ or HND or HNC or equivalent; College or University degree & Other professional qualification. |
| Smoking | 2,3 | 20116 | Never smoked=0, smoked ever/current=1 |
| Systolic blood pressure | 3 | 4080 | Continuous measure, mmHg |
| Ethnicity | 3 | 21000 | Six categories: white; black; Asian; mixed; Chinese; other |
| BMI | 3 | 21001 | Continuous measure, kg/m^2^ |
| Cholesterol | 3 | 30690 | Continuous measure, mmol/l |
| Townsend index | 3 | 22189 | Continuous measure, no specific units |
| Diabetes | 3 | 2443 | Categorical variable with Yes/No answer in response “Diabetes diagnosed by a doctor” |
| Cholesterol lowering medication use | 3 | 6153 | Categorical variable, Yes/No |
| Anti-hypertensive medication use | 3 | 6153 | Categorical variable, Yes/No |

**Supplementary Results**

Supplementary results are shown for all dementia and for the model described in the main paper (which includes all the stated covariates) unless specifically stated in the figure captions.

APOE and HRT interactions, Length of estrogen and HRT interactions, surgery and HRT interactions

eFigure 2 HRT-APOE interaction for risk of incident dementia

*To produce this model we added an interaction term between HRT and APOE denoted as HRT:APOE. For the interaction term HRT is defined as in the rest of the analysis and APOE is defined as those who have 1 or 2 APOE ɛ4, as throughout the paper. A significant interaction term would imply that the combination of use of HRT with having at least one APOE ɛ4 variant has a significant association with dementia.*

*eFigure 3 HRT-length of exposure to natural estrogen interaction for risk of incident dementia*

To produce this model we added an interaction term between length of exposure to natural estrogen and HRT denoted as HRT:Len. est. For the interaction term HRT is defined as in the rest of the analysis and length of estrogen is defined as those who had less than 37 years of exposure to natural estrogen (defined as 1) or 37 or more years of exposure to natural estrogen (defined as 0).

*eFigure 4 HRT- surgery interaction for risk of incident dementia*

 To produce this model we added an interaction term between surgical menopause (“Surgery”) and HRT denoted as HRT:Surgery. For the interaction term HRT and surgical menopause is defined as in the rest of the analysis. The significant interaction term shows that the combination of use of HRT for those who had surgical menopause is a significant association.

Combinations of surgery, length of natural estrogen and APOE status

*eFigure 5 A decision tree with hazard ratios for the association between HRT use and dementia with different stratifications*

The number in each box is the hazard ratio for that stratification. The question in each box leads to the next (additional) stratification. The top of the tree is overall use of HRT, the first split is shown for surgery, the second for APOE ɛ4 status and the third for length of estrogen.

*eFigure 6* *Further decision trees with hazard ratio for the association between HRT use and dementia with different stratifications*

These decision trees show different ordering of the stratifications.

Different definitions of HRT use

eFigure 7 Risk of incident dementia removing women with short term use

Different definition of HRT use for the adjusted model and all-causedementia. Those women who used HRT but started and stopped at the same age were excluded from the analysis instead of being including in no HRT use.

Removing younger women

There are two potential differences as women get older. The first is that younger women are more likely than older women to take HRT after baseline therefore those women would be in the wrong (never used HRT) category in our analysis. There is nothing we can do about this because we cannot gain information about whether those women subsequently started using HRT. However, we can reduce this effect by excluding younger women - effectively we exclude those women who are more likely to then actually go on to take HRT and therefore be in the wrong group in our analysis. By excluding the younger women we should reduce the number of women potentially in the wrong category (never used HRT). In eFigure 5 we see that there are no significant changes which implies that this is not a significant problem. The second is that women were more likely to use HRT in the past (i.e. a women aged 50 in 2000 was more likely to use HRT than a woman of 50 in 2020). Therefore there may be an issue with different age groups having different usage of HRT which may not be correctly corrected for even with our covariates. We can reduce this potential effect by removing younger women from the analysis. We see no notable difference when we do remove younger women, which again implies this is not a significant concern.

*eFigure 8 Risk of incident dementia removing younger women from the analysis*

For each group labelled by 0, 50, 55, 60, 65, all women under that age were excluded from the analysis. For 0 all women were included. For 50 any women under 50 years old were excluded from the analysis. Results shown are for dementia and all menopause status.

Removing women diagnosed near baseline

*eFigure 9 Risk of incident dementia according to HRT use removing women diagnosed with dementia within stated number of years of baseline*

The cutoffs are set so that any women diagnosed within less than that number of years from their baseline assessment was excluded from that group. For example at 2 yrs cutoff all women who were diagnosed less than 2 years after their baseline assessment were excluded from the analysis.

Surgery subtypes

eFigure 10 Risk of incident dementia stratified by surgery subtypes

UK BB asks three questions relating to hysterectomy or bilateral hysterectomy; this figure shows non-HRT use (Reference) compared to HRT use (HRT used) for women who responded to yes to these questions. The first group is Bilateral oophorectomy (answered yes to UK BB ID 3882: “Have you had BOTH ovaries removed?”). (Hysterectomy 1 are women who answered the question “Have you had a hysterectomy (womb removed)?” (UK Biobank ID 3591). Hysterectomy 2 are women who, when asked “Have you had your menopause (periods stopped)?” (UK Biobank ID 2724) answered: “Not sure - had a hysterectomy”. The Hysterectomy 1 or 2 group where women who answered yes to either, or both, of the hysterectomy 1 or hysterectomy 2 questions.

Stratification by early/late menarche and early/late menopause

eFigure 11 Risk of incident dementia stratified by Early/Late Menarche/Menopause for all-menopause

Early menarche is defined as under 14 and later menarche 14 or older. Early menopause is defined as under 45 and later menopause as 45 or older.

*eFigure 12 Risk of incident dementia stratified by Early/Late Menarche/Menopause for natural-menopause*

Early menarche is defined as under 14 and later menarche 14 or older. Early menopause is defined as under 45 and later menopause as 45 or older.

eFigure 13 Risk of incident dementia stratified by Early/Late Menarche/Menopause for surgical-menopause

 Early menarche is defined as under 14 and later menarche 14 or older. Early menopause is defined as under 45 and later menopause as 45 or older.

Different APOE alleles

eFigure 14 Risk of incident dementia for different combinations of APOE

APOE E2E2 are excluded as there are too few to produce a model.

Excluding APOE ɛ2ɛ4 carriers

eFigure 15 Risk of incident dementia excluding APOE ɛ2ɛ4 carriers

HRT start age for AD and non-AD

We provide additional description of Figure 4 a and b here; this same description applies to the supplement figures below. For both the left (A) and right (B) plots the reference group is women who never used HRT. For A (left plot, where the x-axis shows “less than” age) each point shows the hazard ratio of women who started HRT at the age younger than the noted point on the x-axis compared to women who never took HRT. For example at <47 the hazard ratio is around 1.05 which means that the hazard ratio between women who started taking HRT at age 46 or younger (<47) was slightly (and non-significantly) higher for women who started taking HRT at age 46 or younger than those who never took HRT. Once we start considering more women (as we go from the left side of the x-axis towards the right side of the x-axis we include more of the women in the dataset) the hazard ratio drops below 1. Another way of saying this is that women who started taking HRT earlier than around 48 (where the line rises over the x-axis) had an increased risk of dementia compared to women who never took HRT (ignoring confidence intervals for the moment).

For B (right plot) this shows the same as the left plot but for women who started taking HRT at ages above the point on the x-axis. So at >41 this includes most of the women who took HRT (those women who started taking HRT after the age of 41). Moving along the x-axis shows the hazard ratio of women who started taking HRT at later ages compared to those who never took HRT. This never hits statistical significance except for those women who started taking HRT at older than around 56 years where the hazard ratio compared to women who never took HRT becomes greater than 1 (greater risk than those who never took HRT).

eFigure 16 Risk of incident AD by starting age of HRT

**c**

A) Hazard ratio for those that started using HRT before the stated age with the reference those women who never used HRT or used HRT for less than one year. Solid line is the hazard ratio and the shaded area is the 95% confidence intervals. B) Hazard ratio for those that started using HRT after the stated age with the reference those women who never used HRT or used HRT for less than one year. Solid line is the hazard ratio and the shaded area is the 95% confidence intervals. C) The starting age of HRT is split into four groups: those who started before 46, between 46-50 (inclusive), between 51-56 (inclusive) and those who started after 56. As well as all the women who had had menopause (All menopause) there are also stratifications into those who had a natural menopause (no bilateral oophorectomy or hysterectomy) and a surgical menopause (bilateral oophorectomy or hysterectomy).

eFigure 17 Risk of incident non-AD by starting age of HRT

A) Hazard ratio for those that started using HRT before the stated age with the reference those women who never used HRT or used HRT for less than one year. Solid line is the hazard ratio and the shaded area is the 95% confidence intervals. B) Hazard ratio for those that started using HRT after the stated age with the reference those women who never used HRT or used HRT for less than one year. Solid line is the hazard ratio and the shaded area is the 95% confidence intervals. C) The starting age of HRT is split into four groups: those who started before 46, between 46-50 (inclusive), between 51-56 (inclusive) and those who started after 56. As well as all the women who had had menopause (All menopause) there are also stratifications into those who had a natural menopause (no bilateral oophorectomy or hysterectomy) and a surgical menopause (bilateral oophorectomy or hysterectomy).

Window of opportunity for natural and surgical menopause

eFigure 18 Risk of incident dementia by starting age of HRT for women who had a natural menopause

Stratified by women who had a natural menopause.

A) Hazard ratio for those that started using HRT before the stated age with the reference those women who never used HRT or used HRT for less than one year. Solid line is the hazard ratio and the shaded area is the 95% confidence intervals. B) Hazard ratio for those that started using HRT after the stated age with the reference those women who never used HRT or used HRT for less than one year. Solid line is the hazard ratio and the shaded area is the 95% confidence intervals.

eFigure 19 Risk of incident dementia by starting age of HRT for women who had a surgical menopause

Stratified by women who had a surgical menopause. The y-axis scale is different than other equivalent plots.

A) Hazard ratio for those that started using HRT before the stated age with the reference those women who never used HRT or used HRT for less than one year. Solid line is the hazard ratio and the shaded area is the 95% confidence intervals. B) Hazard ratio for those that started using HRT after the stated age with the reference those women who never used HRT or used HRT for less than one year. Solid line is the hazard ratio and the shaded area is the 95% confidence intervals.

Recreation of the main paper plots with no covariates

eFigure 20 Risk of incident dementia stratified by groups

Abbreviations: APOE, apolipoprotein E. All results from a Cox proportional hazards regression not additionally adjusted. The reference for each group is those who never took HRT or took HRT but started and stopped at the same age. The women defined as using HRT had used HRT for at least one year or were still taking HRT at baseline. Natural menopause is women who reported cessation of periods and who had not had a bilateral oophorectomy or a hysterectomy. Surgical menopause are women who had either a bilateral oophorectomy or hysterectomy. No APOE ɛ4 women do not have any ɛ4 alleles. APOE ɛ4 carriers had either one or two ɛ4 alleles. Estrogen length is defined as time between menarche and the earliest of age at menopause, age at hysterectomy or age at bilateral oophorectomy. Shorter estrogen length is less than 37 years of natural estrogen length. Longer estrogen length is 37 years or more of natural estrogen length.

eFigure 21 Risk of incident AD stratified by groups

Abbreviations: APOE, apolipoprotein E. All results from a Cox proportional hazards regression not additionally adjusted. The reference for each group is those who never took HRT or took HRT but started and stopped at the same age. The HRT used women used HRT for at least one year or were still taking HRT at baseline. Natural menopause is women who reported cessation of periods and who had not have a bilateral oophorectomy or a hysterectomy. Surgical menopause are women who had either a bilateral oophorectomy or hysterectomy. No APOE ɛ4 women did not have any ɛ4 alleles. APOE ɛ4 carriers had either one or two ɛ4 alleles. Estrogen length is defined as time between menarche and the earliest of age at menopause, age at hysterectomy or age at bilateral oophorectomy. Shorter estrogen length is less than 37 years of estrogen length. Longer estrogen length is 37 years or more of estrogen length.

eFigure 22 Risk of incident non-AD stratified by groups

Abbreviations: APOE, apolipoprotein E. All results from a Cox proportional hazards regression not additionally adjusted. The reference for each group is those who never took HRT or took HRT but started and stopped at the same age. The HRT used women used HRT for at least one year or were still taking HRT at baseline. Natural menopause is women who reported cessation of periods and who had not have a bilateral oophorectomy or a hysterectomy. Surgical menopause are women who had either a bilateral oophorectomy or hysterectomy. No APOE ɛ4 women did not have any ɛ4 alleles. APOE ɛ4 carriers had either one or two ɛ4 alleles. Estrogen length is defined as time between menarche and the earliest of age at menopause, age at hysterectomy or age at bilateral oophorectomy. Shorter estrogen length is less than 37 years of estrogen length. Longer estrogen length is 37 years or more of estrogen length.

eFigure 23 Risk of incident dementia by starting age of HRT

A) Hazard ratio for those that started using HRT before the stated age with the reference those women who never used HRT or used HRT for less than one year. Solid line is the hazard ratio and the shaded area is the 95% confidence intervals. B) Hazard ratio for those that started using HRT after the stated age with the reference those women who never used HRT or used HRT for less than one year. Solid line is the hazard ratio and the shaded area is the 95% confidence intervals. C) The starting age of HRT is split into four groups: those who started before 46, between 46-50 (inclusive), between 51-56 (inclusive) and those who started after 56. As well as all the women who had had menopause (All menopause) there are also stratifications into those who had a natural menopause (no bilateral oophorectomy or hysterectomy) and a surgical menopause (bilateral oophorectomy or hysterectomy).

Recreation of main paper plots with age, education and smoking status as covariates

eFigure 24 Risk of incident dementia stratified by groups

Abbreviations: APOE, apolipoprotein E. All results from a Cox proportional hazards regression adjusted for: age, education and smoking status. The reference for each group is those who never took HRT or took HRT but started and stopped at the same age. The women defined as using HRT had used HRT for at least one year or were still taking HRT at baseline. Natural menopause is women who reported cessation of periods and who had not had a bilateral oophorectomy or a hysterectomy. Surgical menopause are women who had either a bilateral oophorectomy or hysterectomy. No APOE ɛ4 women did not have any ɛ4 alleles. APOE ɛ4 carriers had either one or two ɛ4 alleles. Estrogen length is defined as time between menarche and the earliest of age at menopause, age at hysterectomy or age at bilateral oophorectomy. Shorter estrogen length is less than 37 years of natural estrogen length. Longer estrogen length is 37 years or more of natural estrogen length.

eFigure 25 Risk of incident AD stratified by groups

Abbreviations: APOE, apolipoprotein E. All results from a Cox proportional hazards regression adjusted for: age, education and smoking status. The reference for each group is those who never took HRT or took HRT but started and stopped at the same age. The HRT used women used HRT for at least one year or were still taking HRT at baseline. Natural menopause is women who reported cessation of periods and who had not have a bilateral oophorectomy or a hysterectomy. Surgical menopause are women who had either a bilateral oophorectomy or hysterectomy. No APOE ɛ4 women did not have any ɛ4 alleles. APOE ɛ4 carriers had either one or two ɛ4 alleles. Estrogen length is defined as time between menarche and the earliest of age at menopause, age at hysterectomy or age at bilateral oophorectomy. Shorter estrogen length is less than 37 years of estrogen length. Longer estrogen length is 37 years or more of estrogen length.

eFigure 26 Risk of incident non-AD stratified by groups

Abbreviations: APOE, apolipoprotein E. All results from a Cox proportional hazards regression adjusted for: age, education and smoking status. The reference for each group is those who never took HRT or took HRT but started and stopped at the same age. The HRT used women used HRT for at least one year or were still taking HRT at baseline. Natural menopause is women who reported cessation of periods and who had not have a bilateral oophorectomy or a hysterectomy. Surgical menopause are women who had either a bilateral oophorectomy or hysterectomy. No APOE ɛ4 women did not have any ɛ4 alleles. APOE ɛ4 carriers had either one or two ɛ4 alleles. Estrogen length is defined as time between menarche and the earliest of age at menopause, age at hysterectomy or age at bilateral oophorectomy. Shorter estrogen length is less than 37 years of estrogen length. Longer estrogen length is 37 years or more of estrogen length

eFigure 27 Risk of incident dementia by starting age of HRT

A) Hazard ratio for those that started using HRT before the stated age with the reference those women who never used HRT or used HRT for less than one year. Solid line is the hazard ratio and the shaded area is the 95% confidence intervals. B) Hazard ratio for those that started using HRT after the stated age with the reference those women who never used HRT or used HRT for less than one year. Solid line is the hazard ratio and the shaded area is the 95% confidence intervals. C) The starting age of HRT is split into four groups: those who started before 46, between 46-50 (inclusive), between 51-56 (inclusive) and those who started after 56. As well as all the women who had had menopause (All menopause) there are also stratifications into those who had a natural menopause (no bilateral oophorectomy or hysterectomy) and a surgical menopause (bilateral oophorectomy or hysterectomy).
